# The interplay between human papillomavirus and vaginal microbiota in cervical cancer development

**DOI:** 10.1186/s12985-023-02037-8

**Published:** 2023-04-19

**Authors:** Kimia Sharifian, Zabihollah Shoja, Somayeh Jalilvand

**Affiliations:** 1 Department of Virology, School of Public Health, Tehran University of Medical Sciences, Tehran, 14155 Iran; 2 Department of Virology, Pasteur Institute of Iran, Tehran, Iran

**Keywords:** Human papillomavirus, Microbiota, Cervical cancer

## Abstract

Over the past few decades, we have grown accustomed to the idea that human papillomavirus can cause tumors. The genetic and environmental factors that make the difference between elimination of viral infection and the development of cancer are therefore an area of active investigation at present. Microbiota has emerged as an important factor that may affect this balance by increasing or decreasing the ability of viral infection to promote. The female reproductive system has its specific microbiota that helps to maintain health and prevent infection with pathogens. In contrast to other mucosal sites, the vaginal microbiota typically has low diversity and contains few *Lactobacillus spp.* which by using high-throughput 16s rRNA gene sequencing, classified into five different community state types. According to emerging information, increased diversity of vaginal microbiota and reduced abundance of Lactobacillus spp. contribute to HPV acquisition, persistence, and development of cervical cancer. In this review, the role of normal female reproductive tract microbiota in health, mechanisms which dysbiosis can cause diseases through interaction with microbes and several therapeutic approaches were addressed.

## Introduction

Cancer is one of the leading causes of death in the world International Agency for Research on Cancer (IARC) reported 19.3 million new cases of cancer and 10 million deaths in 2020 [1]. According to the prediction of World Health Organization (WHO), cancer incidence will be increased by 70% over the next two decades [2]. It is well-documented that almost 15% of cancers caused by several viruses including Human Papillomavirus (HPV), Polyomaviruses, Epstein Barr virus (EBV), Kaposi's sarcoma-associated herpesvirus (KSHV), Hepatitis B virus (HBV), Hepatitis C viruse (HCV), and Human T lymphotropic virus type I (HTLV-1) [3]. Although persistent infection with these viruses can cause several cancers, the most of infected people will never develop cancer. This fact shows that other cofactors are mandatory for development of cancer by viruses [4]. In this regard, studies determined the vital role of microbiota to progress of cancer [5–9].

Microbiota are the range of microorganisms that may be commensal, symbiotic, or pathogenic found in a particular environment. Each site of the human body has particular microbiota which accounts for definite role in human health [10]. Dysbiosis (disruption of microbiota homeostasis) can threaten health condition due to increasing host susceptibility to infections [11, 12]. It is shown that different factors including life style, age, hygiene, sex, host genetic, diet, environmental factors, type of birth delivery, infant feeding methods, diseases, and exposure to antibiotics can affect microbiota. In another word, these factors can help to keep homeostasis and health condition, or they can cause dysbiosis and diseases [13].

## Human papillomavirus and cervical cancer

Human Papillomavirus which belongs to the distinct taxonomic family, the Papillomaviridae and Firstpapillomavirinae subfamily, is a small non-enveloped, epitheliotropic icosahedral DNA virus (60 nm in diameter). The virions consist of a single molecule histone-bound double-stranded circular DNA about 8 kb with eight protein-coding genes and has been divided into three regions: 1–a noncoding regulatory long control region (LCR) which contains promoter, enhancer, and silencer; 2–an early region (E1–E7) which involved in replication and transformation. The HPV E1 and E2 proteins act as origin recognizers of replication; The E2 protein is also a key regulator of viral gene transcription. It is believed that E4, despite its name, is involved in the later stages of the virus life cycle and that E5 may be active in the early and late stages. The E6 and E7 proteins target several negative cell cycle regulators, mainly p105Rb and p53, respectively. During the viral life cycle, E6 and E7 facilitate stable maintenance of viral episomes and stimulate differentiated cells to return to S phase.; and 3–a late region (L1–L2) which encoding capsid proteins and are required for virion assembly. The viral E protein is transcribed from the early promoter while the L protein is mainly transcribed from the late promoter. The viruses are made up of 72 pentameric capsomeres arranged on a surface lattice T = 7. Its capsid consists of 360 copies of the major capsid protein (L1) and 12 molecules of minor protein (L2) [14–16].

Based on DNA sequence homology in the L1 gene, HPV divided into five genera including Alpha, Beta, Gamma, Mu, and Nu [17, 18]. Alpha papillomaviruses infect cutaneous and mucosal epithelium while other genera infect the cutaneous epithelium specially [18, 19]. Till date, more than 220 HPV genotypes have been recognized, among which 40 genotypes can infect anogenital area [20]. However, among these 40 genotypes, 14 genotypes designated as high-risk (HR) HPVs including HPV 16, 18, 31, 33, 35, 39, 45, 51, 52, 56, 58, 59, 66, and 68 can cause several cancers in human including cervix, vagina, vulva, penis, anus, and oropharynx cancer. Low-risk (LR) subtypes are also sometimes found in cervical carcinoma [21]. It is estimated that almost 5.4% of all cancers in human are associated with HPV infection [22–24].

HR-HPV infection is very common in sexually active women and cervical cancer is the most important cancer attributed to HPV [25, 26]. Cervical cancer is the fourth most common cancer and fourth leading cause of cancer death in women globally. About 604,127 new cases and 341,831 deaths from cervical cancer were reported worldwide in 2020 [27]. Cervical lesions are classified according to proportion of cervix infected with dysplasia cells, which includes three cervical intraepithelial neoplasia (CIN) classes including CIN1, CIN2, and CIN3 [14, 19, 28]. According to Bethesda System, precancerous lesions of the cervix are divided into two categories: low grade squamous intraepithelial lesion (LSIL) and high grade squamous intraepithelial lesion (HSIL) [29].

It is shown that the risk of infection with any HPV types is over 80% in a woman life span but the risk of developing invasive cervical cancer is much less than 0.6% [26, 30]. In most healthy women, the virus is cleared by host immune system within 6 month to 3 years [24, 31]. While HPV infection is necessary for development of cervical cancer, it is not sufficient and other cofactors are necessary. Having multiple sexual partners, smoking, long term use of contraceptives and hormonal pills, multiple pregnancies, genetic background, epigenetic changes, weakend immune system, race and vaginal microbiota dysbiosis are noted as cofactors [13, 15, 32, 33].

## Microbiota of female reproductive system

The female reproductive system has its specific microbiota and it can undergo changes during the female life process and menstrual cycle [34]. In contrast to other mucosal site of body that the diversity of microbiota is high (particularly gut mucosa), the diversity of vaginal microbiota in the healthy state is low [30, 35] which a few species of *Lactobacillus* is dominance [36]. It is well-documented that *Lactobacillus gasseri*, *Lactobacillus crispatus*, *Lactobacillus iners*, *Lactobacillus jensenii* or *Lactobacillus vaginalis* are predominant in the vagina and other *Lactobacillus* species, such as *Lactobacillus acidophilus* are not found in the vagina [37, 38]. Predominant vaginal *Lactobacillus* spp. protect vagina against invading pathogens via several mechanisms [39, 40] that mentioned below.

Through using high-throughput 16 s rRNA gene sequencing, the vaginal microbiota has been classified into five different community state types (CST) including: *L. crispatus* (CST I), *L. gasseri* (CST II), *L. iners* (CST III), *L. jensenii* (CST V). CST IV contains a heterogenous group which divided into two subgroups (CST IV-A and CST IV-B).

CST IV-A has the modest proportion of *Lactobacillus* spp. and low proportions of anaerobic bacteria while CST IV-B has higher proportion of *Atopobium*, *Prevotella*, *Parvimonas*, *Gardnerella*, *Megasphera*, *Ruminococcaceae*, *Mobiluncus*, *Sneathia*, and empty of *Lactobacillus* spp. [30, 41, 42]. In healthy women, CST I and V are dominant microbiota. During infection with HPV, CST II prevails and boosts clearance of HPV infection [43–45]. *L. iners* and other species such as *Bacteroides*, *Fusobacterium*, *Veillonela*, *Actinomycetes*, *Bifidobacterium*, *Peptococcus*, *Peptostreptococcus*, *Propionibacterium*, *Staphylococcus aureus*, *Staphylococcus epidermidis*, *Enterococcus faecalis*, *Gardnerella vaginalis*, and *Prevotella bivia* also exist at low proportions [46]. It is worth mentioning that the composition of the vaginal microbiota is dynamic, as there is a frequent alteration from one microbiota to another in the same woman during her lifetime, generally from CST III to IV [26, 42].

The genital microbiota composition can indirectly affect by the gut microbiota (gut-vagina axis) [35, 47, 48]. Oestrobolome is a collection of gut bacteria and their genes adapted to metabolize oestrogen [49]. These bacteria influence the vaginal microbiota content by oestrogen-mediated machinery. They secrete β-glucuronidase and β-glucosidase that lead to deconjugate hepatically conjugated oestrogens and consequently prompts their reabsorption to circulation. This free oestrogen is transported to distal sites such as lower female reproductive system where it binds to its receptors and triggers intracellular signalling that lead to higher glycogen production and other physiological changes such as mucus production and thickening of the epithelium [35]. Regard to this fact that glycogen is the main nutrient consumed by *Lactobacillus* spp., the higher glycogen production lead to *Lactobacillus spp.* growth [35]. The mucus production and thickening of the epithelium can also prevent the entry of HPV to the host cell. It is shown that low diversity of the gut microbiota could negatively affect the vaginal microbiome composition through the oestrobolome [35].

The main mechanisms which *Lactobacillus* spp. protect the female reproductive system include: (i) competition with pathogens for vaginal epithelium adhesion due to steric hindrance or specific blockage of the receptor site, (ii) inhibition of pathogen migration and progression of epithelial integrity by up-regulating tight junction proteins, (iii) prevention of growth and expansion of pathogens by lactic acid production and acidifying vaginal environment, (iv) production of bacteriocins and hydrogen peroxide (H_2_O_2_) which have antimicrobial effect, (v) developing the autophagy of cells infected by pathogens and help their elimination, and (vi) modulation of local defense [36, 45, 50–58].

Lactic acid has two isomers: D-isomer and L-isomer. Vaginal epithelium, *L. iners*, and anaerobes bacteria produce L-isomer of lactic acid while *L. jensenii* produces D-isomer of lactic acid. The amount of L-isomer production by *L. jensenii* could not be detected. *L. crispatus* and *L. gasseri* produce both isomers [59]. High concentration of D-lactic acid produced by *L. crispatus*-dominated microbiota, increase viscosity of vaginal mucus consequently upgrade its virion trapping ability [36, 60].

## Dysbiosis, HPV infection and cervical cancer

**Fig. 1:**
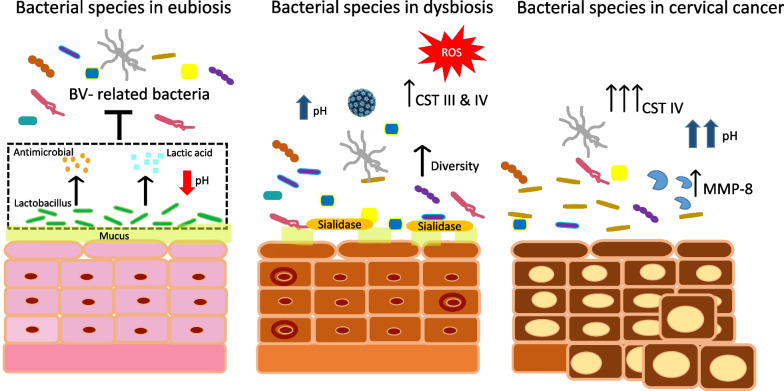
Bacterial species of vagina in eubiosis, dysbiosis, and cervical cancer

The homeostasis of cervicovaginal microbiome is maintained via interaction with the local microenvironment. When this homeostasis is disrupted, leading to a condition known as dysbiosis (Fig. 1). Dysbiosis can be prompted development of cancer through epithelial barrier disruption, metabolic dysregulation, abnormal cellular proliferation, genome instability, chronic inflammation, and angiogenesis [26, 35].

Vaginal *Lactobacillus spp.* is important for maintenance of the cervical epithelial barrier function as it can impede the entry of HPV to the basal keratinocytes throught maintenance of a low pH and bacteriocin production [61]. It is known that *L. crispatus* (CSTI) and *L. gasseri* (CSTII) were the most frequent species in HPV negative women [62] whereas CSTs III and IV are frequently related with the presence of HPV infection and development of premalignant and invasive cervical cancer. It also suggested that *L. gasseri* (CSTII) may be associated with the most rapid clearance of acute HPV infection among HPV positive women [63].

**Fig. 2:**
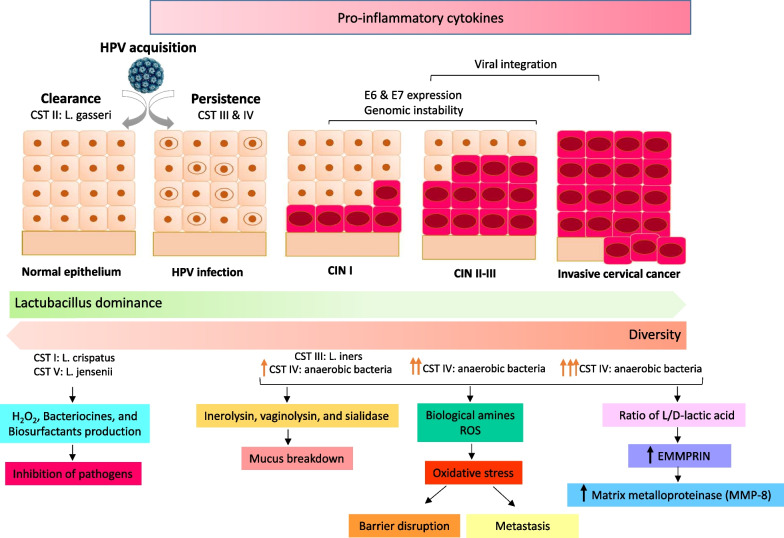
Interpaly between human papillomavirus (HPV), vaginal microbiome and the host. In community state type I (CST I) and CST V, *L. crispatus* and *L. jensenii* are dominant, respectively, which may be protective against HPV acquisition. In CST II, *L. gasseri* is prominent that particularly is associated with clearance of HPV infection. In CST III, *L. iners* is common that is associated with acquisition and persistence of HPV infection and progression to cervical intraepithelial neoplasia I (CIN I). In CST IV, anaerobic bacteria were dominant which is corolated with progression to CIN II/III and consequently to invasive cervical cancer

Among different CSTs, CST III and IV are associated to dysbiosis. *L. iners* is less able to inhibit colonization of pathogens and it can survive in a wide range of pH and other metabolic stress-related situations [42, 64, 65]. *L. iners* produces inerolysin which is a cholesterol dependent pore forming cytotoxin that creates pore in the vaginal epithelium and helps to pathogen entrance [26, 66, 67]. A recent study showed that CST IV subgroup severely correlated with HPV persistence [68]. The highest amount of vaginolysin, another cholesterol dependent cytotoxic protein, is secreted primarily from CST IV especially *G. vaginalis* and then CST III. It can cause cellular lysis, tissue breakdown and may contribute to bacterial vaginosis [69]. Studies had been shown women with CST III and IV microbiota dominance, exhibit a higher ratio of L-to D-lactic acid which cause increase expression of extracellular matrix metalloproteinase inducer (EMMPRIN) which activate matrix metalloproteinase (MMP-8). MMP-8 dissolves intracellular junction by cleaving collagen and alter cervical integrity and facilitate entry of HPV to basal keratinocytes [60]. Moreover, EMMPRIN and MMP-8 are involved in cancer metastasis (Fig. 2) [59, 70].

Vaginal microbiota composition is influenced by several factors such as ethnicity, hormonal changes due to menstruation, age, pregnancy, hygiene habits (vaginal douching), sexual intercourse, and overuse of antibiotics that can alter the vaginal microbiome. Ethnicity is a main factor known to be considerably related to variance in community composition of vaginal microbiota. Indeed, the higher prevalence of *Lactobacillus* spp. dominant microbiota significantly showed in Caucasian and Asian women in comparison to Hispanic and Black women [36]. In African-American women *L. iners* is dominance which can be associated with bacterial vaginosis [26, 42, 43]. Black and Hispanic women displaying polymicrobial vaginal microbiota including *Prevotella*, *Gardnerella*, *Atopobium*, and *Megasphera* species.

This variation may be result of genetic factors that influence host inflammatory immune response or metabolic pathways [71, 72]. It is also hygiene practices such as vaginal douching is distinct in different races, as it is reported twice for Black women than Caucasian women [71]. Vaginal douching by increasing the bacterial diversity may augment the risk of cervical cancer [36].

Bacterial vaginosis (BV), the most prevalent vaginal disorder in reproductive-age women, is a type of dysbiosis which characterized by imbalance and increased microbiota diversity [73]. Vaginal discharge, irritation, fishy odor and increasing vaginal pH, often > 4.5, are BV symptoms [58, 74]. BV has serious consequences including chorioamnitis, spontaneous abortion, preterm delivery, low birth weight, postpartum, endometritis and increased susceptibility to sexually transmitted infections [75]. Women with high diversity of vaginal microbiota are more prone to acquisition of sexually transmitted viral infections including HSV-2, HPV, and HIV [20, 58, 76, 77]. Alpha-papillomaviruses were more common in women with high bacterial diversity than in those with *Lactobacillus* dominant microbiota, according to metagenomic project [78–80]. It is demonstrated *Prevotella* species help to increase diversity and disturb the microbiota homeostasis by providing nutrients for other BV-associated bacteria [81, 82]. Lee’s study showed a plain link between *prevotella* and HPV infection [83]. It is also shown that *Sneathia* spp. was the most common bacteria in women with HPV infection and premalignant lesions, whereas *Fusobacterium* spp. was found to be associated with cervical cancer [62, 84–86].

Regard to metabolomic studies, different metabolomic profiles were found between HPV-positive and HPV-negative individuals. Indeed, the increased levels of biogenic amines and glycogen-related metabolites were shown in CST III and the decreased levels of glutathione, glycogen, and phospholipid-related metabolites in CST IV among HPV-positive than HPV-negative women [87]. Another study was indicated that three lipid molecules including, 3-hydroxybutyrate, eicosenoate, and oleate/vaccinate were enriched in cervical cancer patients [88].

Due to dysbiosis several hallmarks of cancer including barrier disruption, abnormal cellular proliferation, genomic instability, angiogenesis, chronic inflammation, and dysregulation of metabolism can be induced [26]. Oxidative stress due to dysbiosis, generates reactive oxygen species (ROS) which can damage proteins, lipids, and can cause double stranded DNA breaks in HPV episome and host genome, consequently facilitating HPV genome integration and consequently cell transformation [36, 89]. It is well-known that E2 viral gene inhibits the expression of E6 and E7 oncoproteins. However, after viral genome integration, expression of E2 gene is mainly lost result for uncontrolled E6 and E7 expression directs to increased cellular proliferation and decreased apoptosis [26, 30, 36, 90]. E7 protein of HPV also induces angiogenesis [91].

Chronic inflammation is another hallmark of cancer. Some BV associated bacteria such as *Atopobium* can activate the proinflammatory transcription factor nuclear factor-kB (NF-kB), tumor necrosis factor α (TNF α), IL-6, IL-8, and macrophage inflammatory protein 3α (MIP 3α) [92–94]. Furthermore, Other BV-associated bacteria have similar proinflammatory profile with increased IL-1α, β and Ɣ, IL-8, TNF α and granulocyte–macrophage colony stimulating factor (GM-CSF) [30, 95, 96]. Due to inflammatory condition that causes the tissue damage, it may increase the carcinogenic potential of HPV. In the course of DNA damaging, expression of E6 and E7 viral proteins inhibit apoptosis and increase abnormalities leading to cervical cancer [97].

Fusobacteria (*Fusobacterium* and *Sneathia*) and *G. vaginalis* are another BV-associated bacteria which secret sialidase enzyme and cause mucus breakdown, consequently predispose the cervical epithelium to viral infection [83, 98]. *Fusobacterium* spp. likewise activates the WNT signaling pathway by producing its virulence factor, Fad A. WNT signaling pathway is a crucial survival and proliferation pathway which is found in cervical cancer [99, 100].

## Vaginal microbiome modulation approaches

Novel approaches to modulate vaginal microbiota from dysbiotic to optimal *Lactobacillus*- dominant community state could be beneficial and can lead to regress of lesions and improve therapeutic efficacy [35]. Current approaches for modulating vaginal microbiome include probiotics, prebiotics, vaginal microbiota transplantation (VMT), and biofilm disruptors [35, 101, 102].

Probiotics are considered as live microorganisms in the form of supplements or within a food product that when administered in sufficient amounts confer a health benefit to the host [94, 103]. Probiotics include living form of some bacteria species such as *Bifidobacteria*, *Lactobacillus*, and *Streptococci* species which can modify the composition of microbiota, enhance host immune response, used as an auxiliary to antibiotics (Metronidazole, Clindamycin) in BV to improve the vaginal flora, enhance treatment, and impede recurrence. Metronidazole and Clindamycin target the overgrowth of anaerobes. *Lactobacillus rhamnosus* GR-1 in combination with *Limosilactobacillus reuteri* RC-14 increase the *Lactobacillus* dominant vaginal microbiota prevalence [13, 36]. Verhoeven and colleagues evaluated the effects of probiotics on cytological alterations of the uterine cervix and on HPV infection. Fifty one women with HPV + low grade squamous intraepithelial lesion were followed for six months. Twenty four women (intervention group) consumed the Yakult® probiotic that contain *L. paracasei* daily, and 27 women formed the control group. After three months, HPV was cleared in 16% of the subjects (25% of probiotic takers and 7.7% of control group). Up to six months, HPV was cleared in 19% of control groups and 29% of probiotic takers. This study showed that the chance of HPV infection clearance was twice as high in probiotic takers group than in control group [104]. Another study was performed on 117 women with BV or vaginitis with concomitant HPV infection, 60 women consumed probiotics (capsule of *L. rhamnosus* BMX 54) for three months (group 1) and 57 women consumed probiotics for six months (group 2). After the follow up, the chance of HPV clearance in group 2 was twice as high compared to group 1, hence the clearance of HPV infection and regression of cytological changes were greater in group 2 who used the probiotic for a longer period time [105]. In a study, treatment of HPV 16 infected cervical cell line with *Bifidobacterium adolescentis* SPM1005-A revealed lower production of mRNA E6 and E7, suggesting that *B. adolescentis* SPM1005-A may act as a novel curative of virally transformed cells [106].

Prebiotics are non-digestible compounds that induce the growth or activity of beneficial microorganisms which include the fructo-oligosaccharise (FOS) and gluco-oligosaccharide (GOS), sugar alcohols, lactulose, inulin and raffinose [107]. In Cste et al. study, the efficacy of an intravaginal prebiotic gel was investigated. It is shown that the gel effectively promoted the recovery of normal vaginal flora after BV treatment [108]. In a randomized trial intervention on 26 women who were suffering from vaginal infections (control group = 12 and treatment group = 14), were selected for the study. Control group received a standard antifungal treatment and treatment group received Konjac glucomannan hydrolysates (GMH) in addition to standard antifungal treatment. This study showed a reduction of fungal infections in both groups, *Lactobacilli* spp. counts increased in treatment group. Results of study demonstrated the recovery of vaginal health microbiota regard to using of GMH [109]. Pre- and probiotics seem to be a constructive intervention, particularly in the developing countries [110].

Vaginal microbiota transplantation is another treatment option for women with vaginal dysbiosis [111]. VMT should be performed using healthy microbiota which is separated from healthy donor into the vagina of a patient [112]. Because of the potential risks of this procedure such as transfer of antimicrobial-resistant microorganisms, incognito pathogens, and transfer of sperm in vaginal fluid which can result in inadvertent pregnancy, it is essential that accurate inclusion/exclusion criteria and comprehensive testing of donor samples including medical assessment, Whiff test, pH measurement, microscopic evaluation, and next generation sequencing be performed. It is a novel approach under investigating intervention and more studies are needed to determine the efficacy or adverse effects of VMT [35, 111, 113].

## Conclusions

The vaginal microbiota play an important role in the acquisition, persistence, and clearance of HPV in the human vagina. Dysbiosis in vaginal microbiota can promote infection with sexually transmitted pathogens. Most studies were found that HPV infection can also increase the bacterial diversity of vagin in comparison to healthy women leading to higher chance of cervical cancer development. Treatment of vaginal dysbiosis can improve female reproductive tract’s health. In the future, vaginal probiotics, prebiotics, novel antimicrobials, biofilm disruptors, and microbiome implants could be used singly or in combination to restore a healthy local microenvironment to the vaginal microbiome to prevent or reduce the vaginal toxic effects of cervical cancer treatment. However, further studies in this regard are mandatory.

## Data Availability

Data available within the article.

## Abbreviations

HPV: Human papillomavirus
HR: High-risk
LR: Low-risk
IARC: International agency for research on cancer
WHO: World Health Organization
CIN: Cervical intraepithelial neoplasia
LSIL: Low grade squamous intraepithelial lesion
HSIL: High grade squamous intraepithelial lesion
CST: Community state types
BV: Bacterial vaginosis
ROS: Reactive oxygen species
VMT: Vaginal microbiota transplantation

## References

[1] International Agency for Research on Cancer. GLOBOCAN (2020). Estimated cancer incidence. Mor Prevalence Worldwide in.

[2] Sung H, Ferlay J, Siegel RL, Laversanne M, Soerjomataram I, Jemal A (2021). Global cancer statistics 2020: GLOBOCAN estimates of incidence and mortality worldwide for 36 cancers in 185 countries. CA Cancer J Clin..

[3] Schiller JT, Lowy DR (2014). Virus infection and human cancer: an overview. Recent Results Cancer Res Fortschritte der Krebsforschung Progres dans les recherches sur le cancer.

[4] Schiller JT, Lowy DR (2021). An Introduction to Virus Infections and Human Cancer. Recent Results Cancer Res Fortschritte der Krebsforschung Progres dans les recherches sur le cancer.

[5] Vyshenska D, Lam KC, Shulzhenko N, Morgun A, editors. Interplay between viruses and bacterial microbiota in cancer development. Seminars in immunology; 2017: Elsevier. 10.1016/j.smim.2017.05.003 PMC5685535 28602713

[6] Baldridge MT, Nice TJ, McCune BT, Yokoyama CC, Kambal A, Wheadon M (2015). Commensal microbes and interferon-λ determine persistence of enteric murine norovirus infection. Science.

[7] Buchli V, Pearce WB (1974). Listening behavior in coorientational states. J Commun.

[8] Konrad H, Rattenborg C (1969). Combined action of laryngeal muscles. Acta Otolaryngol.

[9] Situnayake R, Thurnham D, Kootathep S, Chirico S, Lunec J, Davis M (1991). Chain breaking antioxidant status in rheumatoid arthritis: clinical and laboratory correlates. Ann Rheum Dis.

[10] Ogunrinola GA, Oyewale JO, Oshamika OO, Olasehinde GI. The human microbiome and its impacts on health. Int J Microbiol. 2020;2020. 10.1155/2020/8045646 PMC7306068 32612660

[11] Aviles-Jimenez F, Yu G, Torres-Poveda K, Madrid-Marina V, Torres J (2017). On the search to elucidate the role of microbiota in the genesis of cancer: the cases of gastrointestinal and cervical cancer. Arch Med Res.

[12] Schwabe RF, Jobin C (2013). The microbiome and cancer. Nat Rev Cancer.

[13] Busnelli M, Manzini S, Chiesa G (2020). The gut microbiota affects host pathophysiology as an endocrine organ: a focus on cardiovascular disease. Nutrients.

[14] Brianti P, De Flammineis E, Mercuri SR (2017). Review of HPV-related diseases and cancers. New Microbiol.

[15] Burd EM (2003). Human papillomavirus and cervical cancer. Clin Microbiol Rev.

[16] Van Doorslaer K, Chen Z, Bernard H-U, Chan PK, DeSalle R, Dillner J (2018). ICTV virus taxonomy profile: Papillomaviridae. J Gen Virol.

[17] De Villiers E-M, Fauquet C, Broker TR, Bernard H-U, Zur HH (2004). Classification of papillomaviruses. Virology.

[18] Della Fera AN, Warburton A, Coursey TL, Khurana S, McBride AA (2021). Persistent human papillomavirus infection. Viruses.

[19] Doorbar J, Egawa N, Griffin H, Kranjec C, Murakami I (2015). Human papillomavirus molecular biology and disease association. Rev Med Virol.

[20] Happel A-U, Varsani A, Balle C, Passmore J-A, Jaspan H (2020). The vaginal virome—balancing female genital tract bacteriome, mucosal immunity, and sexual and reproductive health outcomes?. Viruses.

[21] Molina-Pineda A, López-Cardona MG, Limón-Toledo LP, Cantón-Romero JC, Martínez-Silva MG, Ramos-Sánchez HV (2020). High frequency of HPV genotypes 59, 66, 52, 51, 39 and 56 in women from Western Mexico. BMC Infect Dis.

[22] Shiels MS, Kreimer AR, Coghill AE, Darragh TM, Devesa SS (2015). Anal cancer incidence in the United States, 1977–2011: distinct patterns by histology and behavior. Cancer Epidemiol Prevent Biomark.

[23] Control CfD, Prevention. Cancers associated with human papillomavirus. United States—2011–2015 USCS data brief. 2018(4).

[24] de Martel C, Georges D, Bray F, Ferlay J, Clifford GM (2020). Global burden of cancer attributable to infections in 2018: a worldwide incidence analysis. Lancet Glob Health.

[25] Bosch FX, De Sanjosé S. Chapter 1: Human papillomavirus and cervical cancer—burden and assessment of causality. JNCI Monogr. 2003;2003(31):3–13. 10.1093/oxfordjournals.jncimonographs.a003479 12807939

[26] Castanheira CP, Sallas ML, Nunes RAL, Lorenzi NPC, Termini L. Microbiome and cervical cancer. Pathobiology. 2021:1–11. 10.1159/000511477 33227782

[27] GLOBOCAN U. New Global Cancer Data. 2020.

[28] Anderson C, Lee A, McLaren K, Cairns S, Cowen C, McQueen F (2004). Level of agreement and biopsy correlation using two-and three-tier systems to grade cervical dyskaryosis. Cytopathology.

[29] Ostör A (1993). Natural history of cervical intraepithelial neoplasia: a critical review. Int J Gynecol Pathol.

[30] Kyrgiou M, Mitra A, Moscicki A-B (2017). Does the vaginal microbiota play a role in the development of cervical cancer?. Transl Res.

[31] Sasagawa T, Takagi H, Makinoda S (2012). Immune responses against human papillomavirus (HPV) infection and evasion of host defense in cervical cancer. J Infect Chemother.

[32] Boda D, Docea AO, Calina D, Ilie MA, Caruntu C, Zurac S (2018). Human papilloma virus: apprehending the link with carcinogenesis and unveiling new research avenues. Int J Oncol.

[33] Lin L, Benard VB, Greek A, Hawkins NA, Roland KB, Saraiya M (2015). Racial and ethnic differences in human papillomavirus positivity and risk factors among low-income women in Federally Qualified Health Centers in the United States. Prev Med.

[34] Chen C, Song X, Wei W, Zhong H, Dai J, Lan Z (2017). The microbiota continuum along the female reproductive tract and its relation to uterine-related diseases. Nat Commun.

[35] Łaniewski P, Ilhan ZE, Herbst-Kralovetz MM (2020). The microbiome and gynaecological cancer development, prevention and therapy. Nat Rev Urol.

[36] Mitra A, MacIntyre DA, Marchesi JR, Lee YS, Bennett PR, Kyrgiou M (2016). The vaginal microbiota, human papillomavirus infection and cervical intraepithelial neoplasia: what do we know and where are we going next?. Microbiome.

[37] Younes JA, Lievens E, Hummelen R, van der Westen R, Reid G, Petrova MI (2018). Women and their microbes: the unexpected friendship. Trends Microbiol.

[38] Martin DH, Marrazzo JM (2016). The vaginal microbiome: current understanding and future directions. J Infect Dis..

[39] Ravel J, Gajer P, Abdo Z, Schneider GM, Koenig SS, McCulle SL (2011). Vaginal microbiome of reproductive-age women. Proc Natil Acad Sci USA.

[40] Hickey RJ, Zhou X, Pierson JD, Ravel J, Forney LJ (2012). Understanding vaginal microbiome complexity from an ecological perspective. Transl Res.

[41] Łaniewski P, Herbst-Kralovetz M (2018). Vagina. Encyclopedia of Reproduction.

[42] Romero R, Hassan SS, Gajer P, Tarca AL, Fadrosh DW, Nikita L (2014). The composition and stability of the vaginal microbiota of normal pregnant women is different from that of non-pregnant women. Microbiome.

[43] Brotman RM, Shardell MD, Gajer P, Tracy JK, Zenilman JM, Ravel J (2014). Interplay between the temporal dynamics of the vaginal microbiota and human papillomavirus detection. J Infect Dis.

[44] Shannon B, Yi T, Perusini S, Gajer P, Ma B, Humphrys M (2017). Association of HPV infection and clearance with cervicovaginal immunology and the vaginal microbiota. Mucosal Immunol.

[45] Xu J, Peng J-J, Yang W, Fu K, Zhang Y (2020). Vaginal microbiomes and ovarian cancer: a review. Am J Cancer Res.

[46] Leyva-Gómez G, Prado-Audelo D, María L, Ortega-Peña S, Mendoza-Muñoz N, Urbán-Morlán Z (2019). Modifications in vaginal microbiota and their influence on drug release: challenges and opportunities. Pharmaceutics.

[47] Freitas AC, Hill JE (2017). Quantification, isolation and characterization of Bifidobacterium from the vaginal microbiomes of reproductive aged women. Anaerobe.

[48] Baker JM, Al-Nakkash L, Herbst-Kralovetz MM (2017). Estrogen-gut microbiome axis: physiological and clinical implications. Maturitas.

[49] Kwa M, Plottel CS, Blaser MJ, Adams S. The intestinal microbiome and estrogen receptor–positive female breast cancer. JNCI 2016;108(8) 10.1093/jnci/djw029 PMC5017946 27107051

[50] De Seta F, Campisciano G, Zanotta N, Ricci G, Comar M (2019). The vaginal community state types microbiome-immune network as key factor for bacterial vaginosis and aerobic vaginitis. Front Microbiol.

[51] Kovachev SM (2020). Cervical cancer and vaginal microbiota changes. Arch Microbiol.

[52] Li H, Zang Y, Wang C, Li H, Fan A, Han C (2020). The interaction between microorganisms, metabolites, and immune system in the female genital tract microenvironment. Front Cell Infect Microbiol.

[53] Nardis C, Mosca L, Mastromarino P (2013). Vaginal microbiota and viral sexually transmitted diseases. Ann Ig.

[54] Boris S, Barbés C (2000). Role played by lactobacilli in controlling the population of vaginal pathogens. Microbes Infect.

[55] Chan R, Reid G, Irvin R, Bruce A, Costerton J (1985). Competitive exclusion of uropathogens from human uroepithelial cells by Lactobacillus whole cells and cell wall fragments. Infect Immun.

[56] Reid G, Cook RL, Bruce AW (1987). Examination of strains of lactobacilli for properties that may influence bacterial interference in the urinary tract. J Urol.

[57] Ghadimi D, de Vrese M, Heller KJ, Schrezenmeir J (2010). Lactic acid bacteria enhance autophagic ability of mononuclear phagocytes by increasing Th1 autophagy-promoting cytokine (IFN-γ) and nitric oxide (NO) levels and reducing Th2 autophagy-restraining cytokines (IL-4 and IL-13) in response to Mycobacterium tuberculosis antigen. Int Immunopharmacol.

[58] Torcia MG (2019). Interplay among vaginal microbiome, immune response and sexually transmitted viral infections. Int J Mol Sci.

[59] Witkin SS, Mendes-Soares H, Linhares IM, Jayaram A, Ledger WJ, Forney LJ (2013). Influence of vaginal bacteria and D-and L-lactic acid isomers on vaginal extracellular matrix metalloproteinase inducer: implications for protection against upper genital tract infections. MBio.

[60] Nunn KL, Wang Y-Y, Harit D, Humphrys MS, Ma B, Cone R (2015). Enhanced trapping of HIV-1 by human cervicovaginal mucus is associated with Lactobacillus crispatus-dominant microbiota. MBio.

[61] Mitra A, MacIntyre DA, Marchesi JR, Lee YS, Bennett PR, Kyrgiou M (2016). The vaginal microbiota, human papillomavirus infection and cervical intraepithelial neoplasia: what do we know and where are we going next?. Microbiome.

[62] Audirac-Chalifour A, Torres-Poveda K, Bahena-Román M, Téllez-Sosa J, Martínez-Barnetche J, Cortina-Ceballos B (2016). Cervical microbiome and cytokine profile at various stages of cervical cancer: a pilot study. PLoS ONE.

[63] Brotman RM, Shardell MD, Gajer P, Tracy JK, Zenilman JM, Ravel J (2014). Interplay between the temporal dynamics of the vaginal microbiota and human papillomavirus detection. J Infect Dis.

[64] Macklaim JM, Fernandes AD, Di Bella JM, Hammond J-A, Reid G, Gloor GB (2013). Comparative meta-RNA-seq of the vaginal microbiota and differential expression by Lactobacillus iners in health and dysbiosis. Microbiome.

[65] Macklaim JM, Gloor GB, Anukam KC, Cribby S, Reid G (2011). At the crossroads of vaginal health and disease, the genome sequence of Lactobacillus iners AB-1. Proc Natl Acad Sci.

[66] Pleckaityte M (2020). Cholesterol-dependent cytolysins produced by vaginal bacteria: certainties and controversies. Front Cell Infect Microbiol.

[67] Curty G, de Carvalho PS, Soares MA. The role of the cervicovaginal microbiome on the genesis and as a biomarker of premalignant cervical intraepithelial neoplasia and invasive cervical cancer. Int J Mol Sci. 2019; 21(1) 10.3390/ijms21010222 PMC6981542 31905652

[68] Di Paola M, Sani C, Clemente AM, Iossa A, Perissi E, Castronovo G (2017). Characterization of cervico-vaginal microbiota in women developing persistent high-risk Human Papillomavirus infection. Sci Rep.

[69] Nowak RG, Randis TM, Desai P, He X, Robinson CK, Rath J (2018). Higher levels of a cytotoxic protein, vaginolysin, in Lactobacillus-deficient community state types at the vaginal mucosa. Sex Transm Dis.

[70] Tang J, Wu Y-M, Zhao P, Yang X-M, Jiang J-L, Chen Z-N (2008). Overexpression of HAb18G/CD147 promotes invasion and metastasis via α3β1 integrin mediated FAK-paxillin and FAK-PI3K-Ca 2+ pathways. Cell Mol Life Sci.

[71] Martino JL, Vermund SH (2002). Vaginal douching: evidence for risks or benefits to women’s health. Epidemiol Rev.

[72] Anahtar MN, Byrne EH, Doherty KE, Bowman BA, Yamamoto HS, Soumillon M (2015). Cervicovaginal bacteria are a major modulator of host inflammatory responses in the female genital tract. Immunity.

[73] Chee WJY, Chew SY, Than LTL (2020). Vaginal microbiota and the potential of Lactobacillus derivatives in maintaining vaginal health. Microb Cell Fact.

[74] Brotman RM, Shardell MD, Gajer P, Fadrosh D, Chang K, Silver M (2014). Association between the vaginal microbiota, menopause status and signs of vulvovaginal atrophy. Menopause (New York, NY).

[75] Fethers KA, Fairley CK, Hocking JS, Gurrin LC, Bradshaw CS (2008). Sexual risk factors and bacterial vaginosis: a systematic review and meta-analysis. Clin Infect Dis.

[76] Gosmann C, Anahtar MN, Handley SA, Farcasanu M, Abu-Ali G, Bowman BA (2017). Lactobacillus-deficient cervicovaginal bacterial communities are associated with increased HIV acquisition in young South African women. Immunity.

[77] Shannon B, Gajer P, Yi T, Ma B, Humphrys M, Thomas-Pavanel J (2017). Distinct effects of the cervicovaginal microbiota and herpes simplex type 2 infection on female genital tract immunology. J Infect Dis.

[78] Wylie KM, Mihindukulasuriya KA, Zhou Y, Sodergren E, Storch GA, Weinstock GM (2014). Metagenomic analysis of double-stranded DNA viruses in healthy adults. BMC Biol.

[79] Santella B, Schettino MT, Franci G, De Franciscis P, Colacurci N, Schiattarella A (2022). Microbiota and HPV: the role of viral infection on vaginal microbiota. J Med Virol.

[80] Kwon M, Seo SS, Kim MK, Lee DO, Lim MC. Compositional and functional differences between microbiota and cervical carcinogenesis as identified by shotgun metagenomic sequencing. Cancers (Basel). 2019; 11(3) 10.3390/cancers11030309 PMC6468638 30841606

[81] Pybus V, Onderdonk AB (1997). Evidence for a commensal, symbiotic relationship between Gardnerella vaginalis and Prevotella bivia involving ammonia: potential significance for bacterial vaginosis. J Infect Dis.

[82] Pybus V, Onderdonk AB (1998). A commensal symbiosis between Prevotella bivia and Peptostreptococcus anaerobius involves amino acids: potential significance to the pathogenesis of bacterial vaginosis. FEMS Immunol Med Microbiol.

[83] Lee JE, Lee S, Lee H, Song Y-M, Lee K, Han MJ (2013). Association of the vaginal microbiota with human papillomavirus infection in a Korean twin cohort. PLoS ONE.

[84] Lee JE, Lee S, Lee H, Song YM, Lee K, Han MJ (2013). Association of the vaginal microbiota with human papillomavirus infection in a Korean twin cohort. PLoS ONE.

[85] Łaniewski P, Barnes D, Goulder A, Cui H, Roe DJ, Chase DM (2018). Linking cervicovaginal immune signatures, HPV and microbiota composition in cervical carcinogenesis in non-Hispanic and Hispanic women. Sci Rep.

[86] Mitra A, MacIntyre DA, Lee YS, Smith A, Marchesi JR, Lehne B (2015). Cervical intraepithelial neoplasia disease progression is associated with increased vaginal microbiome diversity. Sci Rep.

[87] Borgogna JC, Shardell MD, Santori EK, Nelson TM, Rath JM, Glover ED (2020). The vaginal metabolome and microbiota of cervical HPV-positive and HPV-negative women: a cross-sectional analysis. BJOG.

[88] Ilhan ZE, Łaniewski P, Thomas N, Roe DJ, Chase DM, Herbst-Kralovetz MM (2019). Deciphering the complex interplay between microbiota, HPV, inflammation and cancer through cervicovaginal metabolic profiling. EBioMedicine.

[89] Schmitt A, Harry J, Rapp B, Wettstein F, Iftner T (1994). Comparison of the properties of the E6 and E7 genes of low-and high-risk cutaneous papillomaviruses reveals strongly transforming and high Rb-binding activity for the E7 protein of the low-risk human papillomavirus type 1. J Virol.

[90] Woodman CB, Collins SI, Young LS (2007). The natural history of cervical HPV infection: unresolved issues. Nat Rev Cancer.

[91] Mesri EA, Feitelson MA, Munger K (2014). Human viral oncogenesis: a cancer hallmarks analysis. Cell Host Microbe.

[92] Doerflinger SY, Throop AL, Herbst-Kralovetz MM (2014). Bacteria in the vaginal microbiome alter the innate immune response and barrier properties of the human vaginal epithelia in a species-specific manner. J Infect Dis.

[93] Libby EK, Pascal KE, Mordechai E, Adelson ME, Trama JP (2008). Atopobium vaginae triggers an innate immune response in an in vitro model of bacterial vaginosis. Microbes Infect.

[94] Libertucci J, Young VB (2019). The role of the microbiota in infectious diseases. Nat Microbiol.

[95] Anderson BL, Cu-uvin S, Raker CA, Fitzsimmons C, Hillier SL (2011). Subtle perturbations of genital microflora alter mucosal immunity among low-risk pregnant women. Acta Obstet Gynecol Scand..

[96] Hedge SR, Barrientes F, Desmond RA, Schwebke JR (2006). Local and systemic cytokine levels in relation to changes in vaginal flora. J Infect Dis.

[97] Motevaseli E, Shirzad M, Akrami SM, Mousavi A-S, Mirsalehian A, Modarressi MH (2013). Normal and tumour cervical cells respond differently to vaginal lactobacilli, independent of pH and lactate. J Med Microbiol.

[98] Borgdorff H, Gautam R, Armstrong SD, Xia D, Ndayisaba GF, van Teijlingen NH (2016). Cervicovaginal microbiome dysbiosis is associated with proteome changes related to alterations of the cervicovaginal mucosal barrier. Mucosal Immunol.

[99] Üren A, Fallen S, Yuan H, Usubütün A, Küçükali T, Schlegel R (2005). Activation of the canonical Wnt pathway during genital keratinocyte transformation: a model for cervical cancer progression. Can Res.

[100] Garrett WS (2015). Cancer and the microbiota. Science.

[101] López-Moreno A, Aguilera M (2021). Vaginal probiotics for reproductive health and related dysbiosis: systematic review and meta-analysis. J Clin Med.

[102] Machado D, Castro J, Palmeira-de-Oliveira A, Martinez-de-Oliveira J, Cerca N (2016). Bacterial vaginosis biofilms: challenges to current therapies and emerging solutions. Front Microbiol.

[103] Ebner S, Smug LN, Kneifel W, Salminen SJ, Sanders ME (2014). Probiotics in dietary guidelines and clinical recommendations outside the European Union. World J Gastroenterol: WJG.

[104] Verhoeven V, Renard N, Makar A, Van Royen P, Bogers J-P, Lardon F (2013). Probiotics enhance the clearance of human papillomavirus-related cervical lesions: a prospective controlled pilot study. Eur J Cancer Prev.

[105] Palma E, Recine N, Domenici L, Giorgini M, Pierangeli A, Panici PB (2018). Long-term Lactobacillus rhamnosus BMX 54 application to restore a balanced vaginal ecosystem: a promising solution against HPV-infection. BMC Infect Dis.

[106] Cha M-K, Lee D-K, An H-M, Lee S-W, Shin S-H, Kwon J-H (2012). Antiviral activity of Bifidobacterium adolescentis SPM1005-A on human papillomavirus type 16. BMC Med.

[107] Collins SL, McMillan A, Seney S, van der Veer C, Kort R, Sumarah MW (2018). Promising prebiotic candidate established by evaluation of lactitol, lactulose, raffinose, and oligofructose for maintenance of a lactobacillus-dominated vaginal microbiota. Appl Environ Microbiol.

[108] Coste I, Judlin P, Lepargneur J-P, Bou-Antoun S. Safety and efficacy of an intravaginal prebiotic gel in the prevention of recurrent bacterial vaginosis: a randomized double-blind study. Obstet Gynecol Int. 2012;2012. 10.1155/2012/147867 PMC3536433 23316237

[109] Tester R, Al-Ghazzewi F, Shen N, Chen Z, Chen F, Yang J (2012). The use of konjac glucomannan hydrolysates to recover healthy microbiota in infected vaginas treated with an antifungal agent. Beneficial Microbes.

[110] Ferlay J, Soerjomataram I, Dikshit R, Eser S, Mathers C, Rebelo M (2015). Cancer incidence and mortality worldwide: sources, methods and major patterns in GLOBOCAN 2012. Int J Cancer.

[111] Lev-Sagie A, Goldman-Wohl D, Cohen Y, Dori-Bachash M, Leshem A, Mor U (2019). Vaginal microbiome transplantation in women with intractable bacterial vaginosis. Nat Med.

[112] Ma D, Chen Y, Chen T. Vaginal microbiota transplantation for the treatment of bacterial vaginosis: a conceptual analysis. FEMS Microbiol Lett. 2019;366(4):fnz025. 10.1093/femsle/fnz025 30715301

[113] DeLong K, Bensouda S, Zulfiqar F, Zierden HC, Hoang TM, Abraham AG (2019). Conceptual design of a universal donor screening approach for vaginal microbiota transplant. Front Cell Infect Microbiol.

